# Inflammation in the Pathogenesis of Arrhythmogenic Cardiomyopathy: Secondary Event or Active Driver?

**DOI:** 10.3389/fcvm.2021.784715

**Published:** 2021-12-20

**Authors:** Viviana Meraviglia, Mireia Alcalde, Oscar Campuzano, Milena Bellin

**Affiliations:** ^1^Department of Anatomy and Embryology, Leiden University Medical Center, Leiden, Netherlands; ^2^Cardiovascular Genetics Center, University of Girona-IdIBGi, Girona, Spain; ^3^Centro Investigación Biomédica en Red de Enfermedades Cardiovasculares (CIBERCV), Madrid, Spain; ^4^Medical Science Department, School of Medicine, University of Girona, Girona, Spain; ^5^Department of Biology, University of Padua, Padua, Italy; ^6^Veneto Institute of Molecular Medicine, Padua, Italy

**Keywords:** arrhythmogenic cardiomyopathy, inflammation, immune cells, inflammatory cytokines, autoimmunity, infectious agents, sudden cardiac death

## Abstract

Arrhythmogenic cardiomyopathy (ACM) is a rare inherited cardiac disease characterized by arrhythmia and progressive fibro-fatty replacement of the myocardium, which leads to heart failure and sudden cardiac death. Inflammation contributes to disease progression, and it is characterized by inflammatory cell infiltrates in the damaged myocardium and inflammatory mediators in the blood of ACM patients. However, the molecular basis of inflammatory process in ACM remains under investigated and it is unclear whether inflammation is a primary event leading to arrhythmia and myocardial damage or it is a secondary response triggered by cardiomyocyte death. Here, we provide an overview of the proposed players and triggers involved in inflammation in ACM, focusing on those studied using *in vivo* and *in vitro* models. Deepening current knowledge of inflammation-related mechanisms in ACM could help identifying novel therapeutic perspectives, such as anti-inflammatory therapy.

## Introduction

Arrhythmogenic cardiomyopathy (ACM) is an arrhythmogenic entity not secondary to ischemic, hypertensive, or valvular heart disease that incorporates a broad spectrum of genetic, systemic, infectious, as well as inflammatory disorders. Therefore, ACM includes mainly arrhythmogenic right/left ventricular cardiomyopathy, cardiac amyloidosis, sarcoidosis, Chagas disease, and left ventricular non-compaction (1). In our review, we focus on the classic form of ACM which is a genetically determined cardiomyopathy caused by heterozygous or compound rare deleterious variants in genes encoding mainly proteins of desmosomes. It is a rare genetic disease associated with malignant ventricular arrhythmias and sudden cardiac death (SCD) (2). The histological hallmark is progressive loss of cardiomyocytes and fibro-fatty replacement in the myocardium, originally described as affecting mainly the right ventricle (RV) (3), but left ventricular (LV), or bi-ventricular involvement are now recognized (1). The estimated prevalence of ACM ranges from 1:2,000 to 1:5,000, with age of diagnosis highly variable (young to elderly), but ACM remains the leading cause of SCD in young individuals, especially in athletes (4). Indeed, SCD often occurs as first manifestation of the disease, even in the absence of cardiac structural abnormalities (5). Phenotypic expression is two-three times more common in males, playing putative key role sex hormones (anti-fibrotic effects of estradiol) or sex-based difference in exercise intensity as a disease modifier (6). Even though ACM has a broad and heterogenous phenotypic spectrum, typically disease progression and precipitation are accompanied by the disruption of the myocardial architecture and fibro-fatty replacement, which leads to heart failure. Therefore, an International Expert Consensus Document has proposed a multi-parametric assessment (Padua criteria) including genetics, tissue characterization by cardiac magnetic resonance, depolarization-repolarization ECG abnormalities, and ventricular arrhythmia features to assist a proper ACM diagnosis (7).

The major molecular mechanism involved in the pathogenesis of ACM is the disruption of mechanical integrity at cell-cell junctions due to defects in the desmosomes; however, desmosomal disarray also result in dysregulation of molecular pathways involved in cell differentiation and proliferation (8). Active myocardial inflammation has been observed in up to 70% ACM post-mortem tissues (9–12), where foci of inflammatory cell infiltrates are found especially in areas with extensive fibro-fatty replacement and contain T-lymphocytes, macrophages, neutrophils, and mast cells (10). However, it is currently unknown whether inflammation is a primary event, actively contributing to the disease phenotype, or a secondary event triggered by cardiomyocyte death (13). Although considerable progress has been made in understanding ACM etiopathogenesis, the exact mechanisms associated with the disease are complex and multi-faceted, so that different theories have been proposed to explain ACM etiology. Here, we focus on the “inflammatory theory” by summarizing and critically discussing the most up-to-date information on inflammation/immune response in ACM, focusing on both *in vivo* and *in vitro* models that are helping researchers unraveling the association between inflammation and ACM pathogenesis.

## Genetics of ACM

The genetics of ACM mostly follows an autosomal dominant pattern with reduced penetrance and variable expressivity (14). However, recessive variants of the disease (Naxos disease and Carvajal syndrome, in which ACM is associated with palmoplantar keratosis and wooly hair) (15, 16) and compound and digenic heterozygosity have also been reported (17). Approximately 60% of ACM patients harbor deleterious rare variants in genes encoding proteins of the desmosome (18, 19), with plakophilin-2 (*PKP2*) alterations accounting for 30–40% of the cases (20) (Table 1). Rare deleterious variants in genes encoding non-desmosomal proteins (Table 1) have been associated with ACM phenocopies, accounting for <5% of ACM cases (8).

**Table 1:** List of ACM-associated genes.

**Gene symbol**	**Protein name**
**Desmosomes**	
*JUP*	Plakoglobin
*PKP2*	Plakophilin-2
*DSP*	Desmoplakin
*DSG2*	Desmoglein-2
*DSC2*	Desmocollin-2
***Area Composita*** **and connexome structure**	
*CTNNA3*	αT-catenin
*CDH2*	Cadherin-2
*SCN5A*	Sodium Voltage-Gated Channel Alpha Subunit-5
*ANK2*	Ankyrin-B
*TJP1*	Tight junction protein 1
*TMEM43*	Transmembrane protein 43
**Cytoskeleton**	
*DES*	Desmin
*LMNA*	Lamin A/C
*TTN*	Titin
*FLNC*	Filamin C
*ILK*	Integrin-linked kinase
**Calcium handling machinery**	
*RYR2*	Ryanodine receptor 2
*PLN*	Phospholamban
**Cell signaling pathways**	
*TGFB3*	Transforming growth factor-β3
*TP63*	Tumor pro tein P63
*PPP1R13L*	Protein phosphatase 1 regulatory subunit 13
*PNPLA2*	Patatin-like phospholipase domain containing 2

It is important to remark the overlap of ACM with other inherited cardiac entities. First, LV-ACM shows similarity with a subset of dilated cardiomyopathy (DCM) whose hallmark is the electrical instability and highly arrhythmogenic episodes (1). In addition, two inherited channelopathies phenocopy ACM: catecholaminergic polymorphic ventricular tachycardia (CPVT) and Brugada syndrome (BrS). CPVT is mainly caused by mutations in the *RYR2* gene, but a causative role of *RYR2* for ACM remains to be clarified (21). Similarly, the link between ACM and BrS, mainly caused by mutations in the SCN5A gene, is still controversial (22, 23).

Guidelines for a proper ACM diagnosis recommend genetic testing (see Taskforce Criteria) (24). However, after a complete genetic analysis in ACM families, large part of rare variants remains classified without a definite role (25), impeding a proper clinical translation in each family in a personalized manner (26). Finally, incomplete penetrance and variable expressivity even in carriers of the same mutation indicate a complex etiology and the existence of unknown mechanisms in which genetic heterogeneity, modifier genes and environmental factors (such as physical exercise) might contribute to the disease onset and progression (27).

## Etiopathogenesis Theories For ACM

Although it is clear that disruption of desmosomes and cell-cell contacts are the main mechanisms leading to ACM onset and progression, several theories have been advanced to explain ACM pathogenesis.

The dysontogenetic theory considers ACM a congenital disease in which the absence of myocardium is the consequence of aplasia or hypoplasia of the RV wall, due to abnormal embryonic development. For this reason, Marcus and Fontaine reported the first comprehensive clinical description of the disease using the term “Arrhythmogenic Right Ventricular Dysplasia” (ARVD) (28). The dysontogenic theory has been finally abandoned since the pathology is now recognized as an acquired cardiomyopathy in which patients typically manifest symptoms from adolescence or later (29). Therefore, the name has been replaced with “Arrhythmogenic Right Ventricular Cardiomyopathy” (ARVC) (9). Nowadays, the increasing evidences of left-dominant and biventricular forms led to the broader term “Arrhythmogenic Cardiomyopathy” (ACM) (30), which encompasses all the phenotypical expressions of the disease.

The degenerative/dystrophic theory has been proposed by Basso and colleagues because of the similarity between ACM and the skeletal muscular dystrophies (9). Progressive loss of ventricular myocardium is determined by myocyte death (apoptosis and/or necrosis) triggered by ultrastructural defects and inflammation followed by abnormal fibro-fatty deposition, which acts as a reparative response to replace dead cardiomyocytes (9, 31). This remains the most comprehensive theory to describe the pathogenesis of ACM.

The myocyte apoptotic theory strictly correlates with the dystrophic hypothesis proposing that programmed cell death, also known as apoptosis, is responsible for progressive myocyte death followed by fibro-fatty replacement (32, 33).

Both dystrophic and apoptotic theories imply that the fibro-fatty replacement of ventricular myocardium is an aberrant reparative response to myocardial loss.

The trans-differentiation theory has been advanced to elucidate the origin of the fibro-fatty deposition, suggesting that cardiomyocytes undergo a reprogramming of their cell fate and a switch into adipocytes as consequence of genetic defects (34). Recently, *in vivo* fate mapping and clonal analysis of murine cardiovascular progenitors demonstrated that a subset of cardiomyocytes and cardiac adipocytes develop from common Isl1+/Wt1+ precursors, thus suggesting a strong developmental relationship between the two lineages (35). However, it is well-known that adult cardiomyocytes exhibit limited de-differentiation ability and therefore, it is unlikely that this hypothesis could exclusively explain the source of fibro-adipose tissue in the ACM heart.

The infiltrative theory proposes other resident cardiac cells as source of adipocytes in the myocardium of ACM patients, such as epicardial cells (36), cardiac progenitor cells of the second heart field (37), cardiac fibro-adipogenic progenitors (38), and cardiac stromal cells (39). Altogether, these studies suggest novel non-contractile cell types as contributors to exaggerated adipocyte accumulation in ACM hearts.

Finally, the inflammatory theory addresses the origin of inflammatory infiltrates observed in ACM patients' myocardium (up to 70%) (12) and the pro-inflammatory mediators detected in their blood (40, 41). The inflammation might be a response to cell death/apoptosis, or to viral infection. Indeed, cardiotropic viruses (i.e., enterovirus and adenovirus) are common findings in the ACM hearts (42). The inflammatory hypothesis is not in contrast with a familial transmission of the disease, since genetic factors may play a role in the susceptibility to infections or may amplify the myocardial damage, leading to a progression and worsening of the disease phenotype (43). However, it remains unclear whether the inflammation occurs as a primary infective/immune mechanism causing myocyte damage or as consequence to degenerative loss of myocytes followed by fibro-fatty deposition (44).

In this review, we will further discuss the inflammatory theory, providing an overview of several mechanisms that have been postulated.

## Role of Inflammation in Cardiovascular Diseases and Heart Failure

Both the innate and adaptive immune responses in the heart are activated by myocardial damage and the inflammatory process plays a crucial role in the progression of cardiac dysfunction in heart failure (45). The activation of the immune system in cardiovascular diseases leads to the expression of pro-inflammatory molecules (cytokines, chemokines, interleukins) and the recruitment of macrophages, mast cells, B cells, and T cells. Under inflammatory conditions, cardiac resident macrophages are joined and sometimes replaced by recruited monocyte-derived macrophages (46). Myocardial injury triggers the recruitment of monocytes that infiltrate the damaged tissue, differentiate into macrophages and proliferate locally (47). The newly expanded macrophage population establishes cross-talk with other cell types in the heart such as cardiomyocytes, endothelial cells or fibroblasts by secreting pro-inflammatory mediators such as tumor necrosis factor α (TNF-α) (48), transforming growth factor-β (TGF-β) (49), interleukins (IL-1β and IL-6) (50) and matrix metalloproteinases (46). The macrophages exposed to inflammatory signals stimulate cardiac fibroblasts and cardiomyocytes to adopt a proinflammatory phenotype (51), which in turn secrete proinflammatory cytokines (IL-1 and IL-6), thus propagating the pre-existing inflammation (52). Macrophages play a dominant role in the activation of inflammatory pathways such as Toll-like receptor (TLR), nuclear factor-κB (NFκβ), MAPK and caspase-1 inflammasome pathways, which are then implicated in the activation of oxidative stress, cytokine release and cardiac injury (53). Several studies have also highlighted the role of inflammatory molecules in myocardial remodeling based on their ability to influence cardiac contractility, cardiomyocyte apoptosis, fibrosis, ultimately leading to cardiac hypertrophy and heart failure (48–50, 54). Specifically, myocardial fibrosis induced by cardiac inflammation results in electrical abnormalities and reduction of nutrient supply toward the myocardium, perpetuating a vicious cycle of fibrosis, myocyte death and inflammation (55). Of note, it cannot be excluded that inflammatory infiltrates themselves may also create an arrhythmogenic substrate, in absence of fibrosis (44).

A recent study demonstrated also a direct role of macrophages in collagen deposition during the fibrotic process of heart repair (56). Indeed, cardiac macrophages directly connect to cardiomyocytes by connexin 43 (CX43) gap junction and are electrically coupled with cardiomyocytes, mainly in the atrioventricular (AV) node (57). Notably, depletion or specific CX43 deletion in murine macrophages led to an impairment of AV node conduction (57). This suggests an unexplored role of resident macrophages in the development of cardiac conduction abnormalities in response to myocardial infarction and heart failure, but also under inflammatory conditions as myocarditis or sarcoidosis. Although myocardial inflammation is present in patients with advanced heart failure, regardless of the initial pathogenesis, inflammation itself can be a primary pathological event in cardiomyopathies (55).

Finally, it is now clear that inflammation could contribute directly to electrical remodeling and the risk of arrhythmia. For example, a canine model of sterile pericarditis demonstrated an association between inflammation and alterations in the amount and distribution of CX43 and connexin 40, which led to impaired conduction and atrial arrhythmia vulnerability (58). Furthermore, an NFκB-mediated mechanism contributed to arrhythmic risk by altering the sodium channel transcriptional regulation in rat neonatal ventricular myocytes and rat embryonic cardiomyocyte cell line H9c2 (59).

Altogether this indicates how a deeper understanding of the role of inflammation and immune system in cardiovascular diseases may elucidate new pathophysiologic mechanisms, identifying potential novel therapeutic targets and strategies to modulate the onset, progression and outcome of cardiac disorders.

## Inflammation in ACM Animal Models

Several transgenic animal models have been used to gain insights into the structural, electrophysiological, cellular, and molecular pathways involved in ACM pathogenesis, including inflammation. Identifying the molecular pathways that might promote or facilitate the inflammatory environment in ACM is one of the biggest challenges in understanding the pathological mechanisms of the disease.

### *In vitro* Rat Models

Several *in vitro* studies pointed to key role of inflammation in ACM pathogenesis. These studies mainly focused on the two main desmosomal genes associated with ACM, namely *JUP* and *PKP2*.

The human *JUP*^2157del2^ mutant plakoglobin was expressed using adenovirus in an *in vitro* model of neonatal rat ventricular myocytes (NRVMs) (60). Beside showing subcellular localization redistribution of plakoglobin and myocyte apoptosis, myocytes expressing *JUP*^2157del2^ released more inflammatory mediators such as TNF-α, IL-6, macrophage inflammatory protein-1 α (MIP-1α), and the chemokine regulated upon activation normal T cell expressed and secreted (RANTES) compared to controls (60). Of note, SB216763, a synthetic small molecule inhibitor of glycogen synthase kinase-3 β (GSK3β), reduced the secretion of inflammatory cytokines (60), thus suggesting a link between abnormal Wnt signaling pathway and inflammation in ACM. These results might open the possibility for a tailored pharmacological treatment directed to the molecular mechanism, even though limited by the adverse effects caused by long-term use of Wnt agonists (55). Indeed, long-term inhibition of GSK3β and its consequential activation of Wnt/β-catenin signaling pathways might have unacceptable adverse consequences, including increased risk of developing cancer (61). Recently, the same *in vitro* model was used to study the role of a master regulator of cellular inflammatory process, the NFκB pathway (62), since activation of GSK3β signaling induces inflammation through NFκB pathway. The NFκB inhibitor BAY 11-7082 reduced inflammatory cytokines production and prevented the development of ACM features *in vitro* (63), supporting the link between abnormal Wnt signaling and inflammation through NFκB pathway. In addition, NRVMs infected with adenovirus containing *Pkp2* knock-down construct, displayed higher expression of fibrotic (*Fn1, Col2A1, Col3A1*) and inflammatory (*Il1a* and *Ccl12*) genes, as well as higher STAT3 and NF-κB transcriptional activity compared to control cells (64), revealing a link between loss of Pkp2 and activation of TGF-β1/p38 MAPK pathway, known to increase inflammation and fibrosis (65). Interestingly, TGF-β1/p38 MAPK signaling inhibition by the small molecule inhibitor (5Z)-7-Oxozeaenol reduced the expression of inflammatory and fibrotic genes (64). Finally, short-term exposure of wild type NRVMs to inflammatory cytokines IL-17 and TNF-α determined loss of junctional plakoglobin signal (40), thus supporting the role of inflammatory cytokines in altering plakoglobin distribution also observed in ACM patients (66).

Taken together, *in vitro* observations in rat models support a role of inflammatory process in the pathogenesis of ACM.

### *In vivo* Mouse Models

Several transgenic mice harboring mutations in human (*JUP* and *DSC2*) and murine (*Jup, Dsg2*, and *Pkp2*) desmosomal genes support the implication of inflammation in ACM pathogenesis (67).

Cardiomyocyte-restricted conditional *Jup* knockout mice developed a phenotype similar to human ACM clinical manifestation with spontaneous ventricular arrhythmia and progressive cardiac dysfunction associated with massive cell death, extensive inflammatory infiltration and fibro-fatty replacement (68). The presence of inflammatory infiltrates in these mice, mainly neutrophils and macrophages, was accompanied by increased expression of pro-inflammatory IL-1β and IL-6 in the myocardium. In line with NRVMs expressing the same mutation, cardiac-specific expression of *JUP*^2157*del*2^ in mice caused arrhythmia, deterioration of cardiac function, redistribution of intercalated disc proteins and signs of fibrosis, apoptosis, and focal areas of inflammatory infiltrates (69). In these mice, SB216763 restored cardiac function, with relocalization of proteins at cell-cell junctions and no histological evidence of myocardial fibrosis or inflammatory cell infiltrates. Similar results were confirmed in a different transgenic mouse model with homozygous knock-in of mutant *Dsg2*, in which loss of exons 4 and 5 causes a frameshift mutation and premature termination of translation (*Dsg2*^*mut*/*mut*^) (69). These results confirm that GSK3β inhibition can rescue signs of inflammation together with other features of ACM phenotype in two different transgenic mouse models, emphasizing the tight link between inflammation and ACM. The same *Dsg2*^*mut*/*mut*^ mouse model was used to corroborate the results obtained *in vitro* about the role of NFκB signaling in ACM pathogenesis. *Dsg2*^*mut*/*mut*^ mice developed arrhythmia and progressive ventricular contractile dysfunction associated with extensive myocardial cell death, fibrosis, and inflammation (63). Multiple inflammatory cytokines, including IL-1β, TNF-α, and monocyte chemoattractant protein-1 (MCP1), were expressed in both cardiomyocytes and infiltrating inflammatory cells. Sections of myocardium from *Dsg2*^*mut*/*mut*^ mice showed the presence of inflammatory infiltrates, both macrophages (CD68+ cells) and T-cells (CD3+ cells). BAY 11-7082 treatment improved cardiac function, reduced both cytokine levels and the number of infiltrating inflammatory cells, thus supporting NFκB inhibition as potential therapeutic option *in vivo* (63). Inflammatory response was also reported in other mouse models carrying different mutations in *DSG2*. Transgenic mice with cardiac specific overexpression of *Dsg2*^*N*271*S*^ (corresponding to human mutation *DSG2*^*N*266*S*^) exhibited spontaneous ventricular arrhythmia and sudden death, impaired cardiac function and myocardial damage triggered by massive myocardial necrosis (70). Inflammatory infiltrates of neutrophils and macrophages were observed following myocyte cell death. Similar findings were reported in transgenic mice bearing a cardiac specific deletion of the adhesive extracellular domain in murine Dsg2 protein (71, 72).

Recently, the inflammatory cellular response has been dissected at the different stages of ACM in two *Dsg2* mutant mouse strains, one expressing a truncated Dsg2 protein and another being a cardiomyocyte-specific *Dsg2* knock-out (73). Distinct immune cell infiltrates and chemokine signaling were specifically involved at the different stages. During the early stage of the disease, necrotic cardiomyocytes triggered an inflammatory response characterized by neutrophils recruitment with few macrophages, T cells and by increased gene expression of inflammatory chemokines *Ccl2/Ccr2, Ccl3/Ccr5*, and *Cxcl5/*Cxcr2. The acute disease progression was instead associated with tissue remodeling, formation of collagenous scars and changes in immune cell types (neutrophils were gradually replaced by macrophages and T cells in the scar) and inflammatory chemokine gene expression (upregulation of *Cx3cl1/Cx3cr1, Ccl2/Ccr2, and Cxcl10/Cxcr3, Tnfa* and *Il1b*). During the chronic stage of the disease, inflammatory cells (macrophages and T cells) and cytokine production (*Ccl12, Cx3cl1, Tnfa*, and *Il1b*) persisted within mature scars, although at lower level than in the acute phase.

Transgenic mice with cardiac specific overexpression of human wild-type *DSC2* also developed severe cardiomyopathy characterized by fibrotic and inflammatory remodeling (74). Gene expression analysis revealed the activation of several inflammatory pathways such as cytokine-cytokine receptor interaction, chemokine signaling pathway or Toll-like receptor signaling. In particular, different chemokines (e.g. *Ccl3*) and their receptors (*Ccr2*), toll-like receptors (*Trl9*), interleukins (*Il6, Il33*), and their receptors (*Il7r*) were upregulated in the mutant hearts. The activation of inflammatory response shown by gene expression analysis was confirmed by immunohistochemistry displaying higher signal for CD11b, a molecular marker of macrophages. Recently, transcriptome analysis of adult cardiomyocytes derived from cardiac-specific *Pkp2*-knockout mice revealed a link between *Pkp2* transcript abundance and the expression of genes involved in inflammatory and immune response (75). Notably, the transcriptional changes in *Pkp2*-knockout myocytes mirrored the presence of cell infiltrates positive for T-cell marker CD45 and for neutrophil marker Ly-6G/Ly-6C. These results suggested that endogenous *Pkp2* deficiency is able to regulate the expression of genes involved in inflammatory and immune response, even in the absence of exogenous trigger, as pathogens.

Both primary cardiomyocytes and transgenic animal models have the advantage of making it possible to control for both environmental and genetic factors, circumventing the limitations associated with restricted availability of human cardiac samples, individuals' different genetic background and the limited number of patients carrying the same deleterious variant.

However, the limitation of animal models is mainly related to their non-human origin. Therefore, human samples need to be tested to confirm that findings obtained with *in vivo* animal models accurately reflect the human ACM pathobiology.

## Inflammation in ACM Human Samples

In this section, we summarize the observations of inflammatory process in ACM patients obtained by the evaluation of myocardial samples from autopsy and endomyocardial biopsies, *in vitro* model based on human induced pluripotent stem cell (hiPSC)-derived cardiomyocytes (hiPSC-CMs) and circulating inflammatory biomarkers from serum/plasma.

### Inflammatory Infiltrates (T-Cells) in Explanted Hearts and Endomyocardial Biopsies

**Figure 1:**
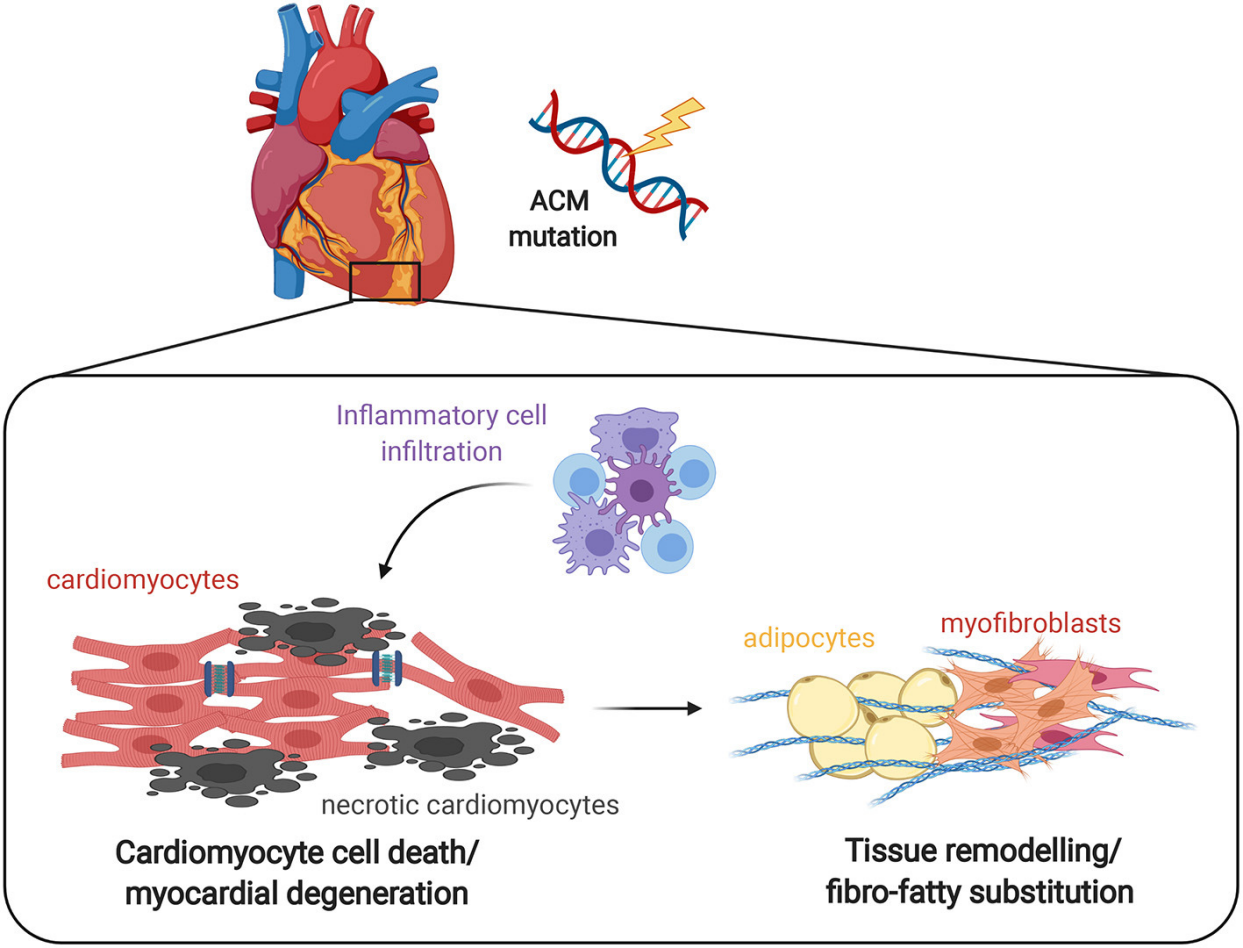
Effect of inflammation on ACM heart. The progressive myocyte death (apoptosis and/or necrosis) and myocardial degeneration due to desmosomal defects leads to the accumulation of inflammatory cell infiltrates, that ultimately results in tissue remodeling by abnormal fibro-adipose deposition as a reparative response to replace loss of cardiomyocytes. It remains to be clarified whether the inflammatory process is the first event that determines the death of cardiomyocytes and the consequent repair process or rather is a reactive phenomenon.

Histological analysis of explanted hearts, post-mortem samples, and endomyocardial biopsies provided evidence of infiltrating inflammatory cells in ACM human cardiac tissues (12) (Figure 1). Autoptic studies identified myocardial inflammation as T- cell infiltrates, consisting of CD45+ lymphocytes (9). Patchy inflammatory infiltrates in proximity to necrotic or degenerative myocytes were detected in ~70% of all the ACM cases (9, 11). Specifically, the inflammatory infiltrates were found mainly in association with fibro-fatty replacement and LV involvement (75%). On the contrary, inflammation was observed only in 30% of ACM patient with the right ventricular form (11). A correlation between the presence of multifocal T-lymphocyte infiltrates and the severity of structural alterations (such as biventricular disease and extended fatty accumulation in the atria) was also confirmed in an independent study (10).

One study described that ALVC often showed patterns of sub-epicardial scarring with a predilection for the left ventricular inferior and lateral walls, similar to that seen following myocarditis (76). Another study reported increased levels of macrophage-related markers (CD68, CD163, CD45) in endomyocardial biopsies from patients with biventricular ACM (77). Of note, a recent study on a subset of patients carrying pathogenic rare alterations in *DSP* with severe LV predominant cardiomyopathy showed distinct LV myocardial injury associated with myocardial inflammation (78). It is worth mentioning that, even in the absence of inflammatory infiltrates, higher expression of inflammatory cytokines IL-17 and TNF-α were detected in ACM post-mortem myocardial samples and endomyocardial biopsies compared to autoptic samples from individuals with no history of cardiac diseases (40). The first report on children with clinical suspicion of myocarditis who had genetic testing for ACM showed that the disease can present as recurrent myocarditis-like episodes with cardiac magnetic resonance evidence of myocardial inflammation despite absent infectious trigger, suggesting an active “hot phase” of ACM that may lead to disease progression (79).

Altogether these reports suggest that inflammatory process might have a key role in the modulation of disease severity. Further studies are needed to investigate inflammatory molecular pathways associated with progression of the disease and induction of malignant arrhythmias.

### Human ACM *in vitro* Models and the Role of Inflammation

Soon after their discovery (80), hiPSCs were differentiated into cardiomyocytes (hiPSC-CMs) to model cardiac diseases, including ACM (81–83). The signaling pathway of NFκB was investigated in *PKP2*-mutated hiPSC-CMs from a patient with ACM (63). Under basal conditions, ACM hiPSC-CMs displayed higher expression and nuclear accumulation of phosphorylated transcription factor RelA/p65, an essential transcription factor responsible for heterodimer formation and nuclear translocation of the NFκB complex (62), indicating the activation of this pathway. Furthermore, similarly to NRVMs transfected with human *JUP*^2157del2^ (60) and transgenic *Dsg*2^mut/mut^ mice (63) as described above, ACM hiPSC-CMs expressed and secreted larger amounts of inflammatory cytokines such as IL-1β, IL-12, interferon-γ, TNFα, and RANTES (63). In addition, several chemotactic molecules were greatly increased, including cytokine-induced neutrophil chemoattractant (CINC-1), macrophage colony-stimulating factor (M-CSF) and the neutrophil chemoattractant LIX. Interestingly, BAY 11-7082 treatment in ACM hiPSC-CMs reduced phospho-RelA/p65 nuclear accumulation and cytokines levels (63). These results support a signature of immune activation in a pure population of ACM hiPSC-CMs (>95%) under the control of NFκB signaling, independently of the presence of circulating or tissue-resident inflammatory cells, such as macrophages or lymphocytes. HiPSC-CMs overcome the disadvantage of limited availability and short *in vitro* life-span of primary human cells, as well the disadvantages of species-specific differences of animal models. However, the immaturity of hiPSC-CMs represents their main limitation, even though solutions to maturation are starting to emerge (84).

### Pro- and Anti-inflammatory Mediators' Imbalance in Serum/Plasma of ACM Patients

Several studies have looked for inflammatory cytokines in the blood of ACM patients, in an attempt to identify potential circulating inflammatory biomarkers (85). Interestingly, higher levels of pro-inflammatory cytokines IL-1β, IL-6, and TNF-α were reported in ACM patients, while the anti-inflammatory cytokine IL-10 was not significantly different compared with controls groups (41). Of note, circulating IL-1β correlated positively to regional myocardial inflammation as assessed by ^67^Ga scintigraphy, even though statistical significance was not reached, probably due to the small number of individuals (8 patients) involved in this study. In another study, ACM samples displayed increased levels of pro-inflammatory cytokines including IL-6 receptor (IL-6R), IL-8, MCP1, macrophage inflammatory protein1b (MIP-1β), and TNF-α receptor types 1 and 2 (TNFR1 and TNFR2) compared with controls (40). In addition, the anti-inflammatory cytokine IL-1 receptor 2 (IL-1R2) was significantly reduced in ACM patients compared with controls (40), thus suggesting imbalance between pro- and anti-inflammatory mediators in ACM. Notably, elevated levels of TNF-α and IL-1β have been associated with the production of inducible nitric oxide synthase (iNOS) (86) that can ultimately lead to apoptosis and progressive loss of myocardium followed by fibro-adipose deposition (87).

Higher levels of C-reactive protein (CPR) were also detected in the plasma of individuals affected by ACM right after ventricular arrhythmia (VA) compared to patients with idiopathic ventricular tachycardia (88), suggesting a correlation between inflammation and arrhythmic events, even in the absence of morpho-functional anomalies of the right ventricle.

Although these studies are crucial for potential identification of circulating biomarkers specific for ACM, no diagnostic significance for elevated cytokine levels has been proved yet, especially because unbalanced inflammatory mediators have been described in several cardiovascular diseases (89–91). Further investigation is required together with validation in larger cohorts of ACM patients.

### Autoimmune Response in Serum of ACM Patients

An autoimmune etiology has been recently proposed for ACM pathogenesis (92, 93), hypothesizing that mutated proteins with unmasked cryptic epitopes (94) might be released into the intercellular space and/or circulation as a result of desmosomal disruption and myocardial damage, thus stimulating autoantibodies production (92). A recent study reported the presence of autoantibodies against desmoglein 2 (DSG2) in the serum of ACM patients, while these were absent in healthy controls and individuals affected by other genetic cardiomyopathies (92). DSG2 antibody density measured by Western blot or ELISA correlated with disease severity estimated by the occurrence of premature ventricular contractions within the ACM group. The presence of serum anti-heart autoantibodies (AHAs) and anti-intercalated disk autoantibodies (AIDAs) has been recently detected in 85% of familial ACM cases and in 45% of sporadic ACM cases, including some healthy relatives. On the contrary, autoantibodies were absent in the control group, including individuals with non-inflammatory cardiac diseases, ischemic heart failure, and healthy donors (93). Serum levels of AHAs and AIDAs were also associated with disease severity in ACM patients evaluated as chest pain, palpitation, lower ventricular ejection fraction and ICD implantation (93).

Although these results are promising in providing new tools for prognosis or risk stratification, larger and independent cohorts of patients are needed to clarify whether the autoantibodies may predict the development and progression of the disease. Further investigation is required to elucidate the molecular mechanisms of autoimmunity and its specificity as a test for ACM, in order to discriminate among other inflammatory myocardial diseases such as myocarditis or sarcoidosis.

### The Overlap of ACM With Myocarditis and Cardiac Sarcoidosis

During the past few years, advancements in molecular, clinical and instrumental diagnosis of ACM have been made. The diagnostic criteria defined by the International Task Force (ITF) have been updated in 2010 in order to increase sensitivity, still maintaining specificity (24). However, the clinical diagnosis is often challenging due to the broad spectrum of phenotypes which includes left-dominant disease variants and the absence of a family history in about 50% of ACM patients (95). For this reason, an international expert report has been published in 2019 to critically review the current ITF diagnostic criteria, recommending potential areas of improvements for a more appropriate clinical use in differential diagnosis (96). Notably, other inflammatory cardiac diseases such as myocarditis (97–99) and cardiac sarcoidosis (100–102) may occasionally phenocopy ACM, resulting in misdiagnosis (103). Inflammation could represent the pre-phenotypic/early stage of ACM in a subgroup of patients, showing an uncommon clinical presentation defined as “hot phase,” characterized by acute chest pain and/or release of myocardial enzymes (104). In this view, strict follow-up and differential diagnosis are required to exclude myocardial infarction and myocarditis. A recent study described a cohort of 12 young female patients with an initial clinical presentation of myocarditis syndrome (chest pain, troponin elevation) who were subsequently diagnosed with ACM (103). These findings highlight that these patients have distinct clinical and genetic features including female predominance, LV involvement and pathogenic variants in *DSP* gene (103). Specifically, genetic testing was essential for the ACM diagnosis in this cohort, suggesting the importance of genetic analysis in myocarditis patients. Non-invasive methods such as electrocardiogram, Holter monitoring, echocardiography and cardiac magnetic resonance, might be insufficient to discriminate among inflammatory diseases mimicking ACM, emphasizing the need for a more specific diagnosis. Endomyocardial biopsy is more invasive but revealed myocarditis in 30–50% of individuals originally classified as ACM using non-invasive criteria (97, 98). Anyways, myocarditis reflects an active phase of inflammation in ACM, leading to changes in the phenotype and to an abrupt complication of the disease.

Several case reports of cardiac sarcoidosis mimicking ACM have been described as well (105, 106). Cardiac sarcoidosis was found in 15% of patients meeting criteria for ACM only after endomyocardial biopsy evaluation (102). Interestingly, reduction of plakoglobin signal at cardiomyocyte cell-cell junctions was also reported in patients with sarcoidosis and giant cell myocarditis (40), associating for the first time these highly arrhythmogenic inflammatory diseases with a specific alteration of intercalated disks previously only observed in ACM (66).

These findings suggest a potential new mechanistic link between ACM and sarcoidosis/myocarditis, in which inflammatory cytokines might promote the disruption of desmosomal proteins, thus explaining the clinical similarities among those diseases (40).

### The Role of Infectious Agents in ACM Pathogenesis

Several infectious agents such as cardiotropic viruses, bacteria and protozoans, have been frequently found in ACM patients. Together with the common findings of inflammatory infiltrates in the myocardium, these observations suggest a possible etiologic role for infectious agents in the onset and the progression of ACM.

Cardiotropic viruses have been often identified in the myocardium of ACM patients and, among them, enteroviruses were the first to be investigated in association with ACM based on results obtained from a mouse model. Indeed, BALB/c mice inoculated with Coxsackie virus B3 developed clinical manifestations similar to ACM, including severe myocarditis limited to the right ventricle, myocardial cell death and fibrosis, and mononuclear cell infiltration (107). Several studies reported the detection of coxsackieviruses type B more frequently in ACM endomyocardial biopsies compared to patients with non-inflammatory cardiac disorders, or undergoing transplantation for end-stage congenital diseases or autoptic samples from individuals with no history of cardiac diseases (42, 108, 109). On the contrary, enteroviral genome was not detected in a study where the myocardium of 20 ACM patients was analyzed (110). The discrepancy of these results may relate to technical issues such as low sensitivity of PCR, degradation of viral RNA during extraction or might reflect different patient selection (43). Other cardiotropic viruses such as adenovirus (42), cytomegalovirus, hepatitis C virus, and parvovirus B19 have been also detected in ACM sporadic forms (43). However, the role of viruses in the pathogenesis of ACM remains an unsolved issue. Further investigations will help to clarify whether viruses are true etiological agents contributing to the disease or whether viral infection is a consequence of the increased vulnerability of diseased myocardium.

Similar to cardiotropic viruses, several other infectious agents including protozoans and bacteria have been associated with ACM such as *Trypanosoma* cruzi (111), *Mycoplasma* (112), and *Bartonella henselae* (113, 114), a bacterial agent associated with endocarditis.

Notably, patients diagnosed with non-familial forms of ACM revealed a significantly elevated IgG antibody titres to *Bartonella henselae* compared to healthy blood donors (113). Although of interest, the possible causal relationship between *Bartonella henselae*-induced myocarditis and non-familial cases of ACM was based only on this single study and therefore larger patient cohorts need to be studied. Also, further validation of serological findings with *Bartonella henselae* detection in endomyocardial biopsies of ACM patients is required.

### Link Between Genetic Defect in ACM and Viral Infection Susceptibility: A Potential Genetic Predisposition?

The frequent detection of cardiotropic viruses in ACM patients suggested that viral infections might be relevant environmental factors for the progression of the disease and its adverse clinical outcomes. However, this hypothesis does not conflict with the familial incidence of ACM since genetic factors may influence the susceptibility to infection (43). In the setting of pathogenic alterations in genes encoding desmosomal proteins, a genetically defective myocardium may create an environment more vulnerable to an infectious agent, leading to the amplification of myocardial inflammation, severe myocyte damage and subsequent precipitation of the disease (115, 116). The first study establishing a link between genetics and viral susceptibility in ACM patients showed that family members carrying the same causative rare variant in the *DSP* gene seemed to be particularly vulnerable to develop viral myocarditis (116). Recently, some reports have also suggested the relation between *DSP* mutations and a family history of recurrent myocarditis (117, 118). In another study, genetic analysis on three cases of myocarditis-related SCD and their family members revealed the presence of rare novel variants predicted as deleterious and potentially pathogenic in ACM-related genes (*PKP2, DSP DSC2*, and *TTN*) (115), also including *DSP* as a crucial gene involved. Taking all data into account, *DSP* cardiomyopathy was proposed as a distinct form of ACM, characterized by episodic myocardial injury, left ventricular fibrosis that precedes systolic dysfunction, and a high incidence of ventricular arrhythmias (78). In addition, acute myocarditis should be considered as an additional criterion for ACM in diagnosed families (119). A recent study suggested clinical myocarditis as an initial ACM presentation proposing that these patients have distinct characteristics including female gender, LV involvement and *DSP* gene variants (103).

Collectively, all the data reinforces the use of genetic testing in patients with a family history of cardiomyopathy or SCD who experienced acute myocarditis. The extension of this intriguing hypothesis to other deleterious variants associated with ACM remains to be supported by further genetic studies on larger cohorts of patients with myocarditis. Another theory linking genetic factors with viral susceptibility suggests that pathogenic variants in unrelated-ACM genes encoding cellular receptors or immune system components may increase the vulnerability to infectious agents inducing myocarditis (43). Recently, deleterious variants in genes associated with cardiomyocyte integrity have been reported in a cohort of children with myocarditis, progressing more rapidly and showing a more severe outcome (120).

In this view, viral infection might be considered the trigger necessary for myocardial apoptosis directly mediated by the virus or indirectly by inflammation, thus leading to progressive myocyte loss and consequent fibro-fatty replacement (43). However, this theory remains to be demonstrated by further investigation.

## Role of Exercise and Immune Response in ACM

Several epidemiological studies showed the benefit of exercise and physical activity for the health in the general population, especially limiting the aging process and preventing the incidence of cardiovascular diseases, heart failure, and many chronic disorders (121, 122). An interplay between physical activity and immune response has been proposed (123) and growing evidence from animal and human studies indicate that intense exercise promotes the production of pro-inflammatory cytokines (TNF-α, IL-1β, and IL-6) (124) and the mobilization and the functional capacity of lymphocyte population, mainly controlled by adrenergic signaling (125, 126). This indicates that the immune system is stimulated in response to exercise. At the same time, adverse effects exerted by physical activity are well-known in the context of life-threatening arrhythmias and SCD in several inherited arrhythmogenic conditions (127), including ACM (128), so that changes in lifestyle and refrain from competitive sports is one of the first recommendations in ACM patients.

In ACM patients, due to the genetically determined fragility of desmosomes, the mechanical stretch of myocytes during endurance exercise may favor cell injury and accentuate apoptosis, the initial phase in the remodeling process, by initiating an inflammatory response, myofibroblast activation, and myocardial scar formation (129). Specifically, the role of exercise as a trigger or even precipitating factor in the development and progression of ACM has been recognized by evidences in both mouse models (130, 131) and clinical studies in ACM patients with (132, 133) and without (129, 134) deleterious variants in desmosomal genes. The association of endurance exercise and ACM pathogenesis in the setting of desmosome deleterious variants was established using transgenic mouse model with heterozygous plakoglobin knock out (*Jup*^+/−^), demonstrating that the development of right ventricular dysfunction and arrhythmias were accelerated by endurance training in *Jup*^+/−^ mice (131). A recent study showed that *Dsg2*^*mut*/*mut*^ mice undergoing chronic physical exercise exhibited myocardial dysfunction, myocyte apoptosis and necrosis, myocardial inflammation and fibrosis, and premature exercise-induced sudden death (130). Of note, exercised *Dsg2*^*mut*/*mut*^ myocytes displayed release of nuclear high mobility group box-1 (HGMB1), an endogenous “danger signal” that works as chemotactic molecule, inducing the activation of the immune response and the recruitment of immune cells (135). In line with this, exercised *Dsg2*^*mut*/*mut*^ mice showed high number of nuclear HMGB1^+^ positive cells, corresponding to inflammatory infiltrating cells surrounding necrotic myocytes (130).

In a clinical study, the risk of developing ventricular arrhythmia and heart failure positively correlated to the intensity and frequency of exercise in ACM patients carrying desmosomal deleterious variants (132). It is clear that myocardium with defective desmosomes might be more susceptible to cell-cell detachment and death, but also to impairment of the electrical coupling and consequent pathological remodeling when exposed to excessive mechanical load during exercise (136). Notably, “gene-elusive” ACM patients required more intense physical activity to exacerbate and accelerate the disease phenotype compared to desmosomal deleterious variants carriers (134), thus suggesting more beneficial effects from exercise reduction in the patients with mutations in genes encoding desmosomal proteins (129). A new potential link has been identified between the activation of immune response and desmosomal perturbation in the heart of ACM patients (40) that might harmonize the adverse impact of intense exercise in both patients with and without desmosomal deleterious alterations. Wild-type NRVMs exposed to pro-inflammatory cytokines (IL-17, IL-6, and TNFα) displayed abnormal internalization of plakoglobin, mimicking the disruption of desmosomes observed in ACM but also in the other inflammatory cardiac conditions which can phenocopy ACM as discussed above, namely sarcoidosis and giant cell myocarditis (40). In this view, the stimulation of the immune response and the production of pro-inflammatory mediators triggered by intense exercise might have a negative impact on desmosomal stability and integrity in general, but this would accelerate the development and progression of ACM. Although this is an intriguing hypothesis, it remains to be determined whether the immune response enhanced by physical activity might be the mediator for the altered distribution of desmosomal proteins at the intercalated disks in ACM. Future studies are required to provide novel insights in the molecular mechanisms underlying the role of exercise in the detrimental progression of ACM.

Future studies are required to provide novel insights into the molecular mechanisms underlying the role of exercise in the detrimental progression of ACM. The next challenge is to translate all the results in the field of exercise to the clinics. In particular it will be necessary to better clarify the definition of “intense/strenuous exercise” in patients, to be able to provide clear, and maybe patient-tailored, recommendation to balance between a good quality of life and the risk of sudden cardiac death (137, 138).

## Mitochondria-Mediated Inflammation in ACM

Several reports demonstrated a correlation between oxidative stress and inflammation in many cardiovascular disorders such as hypertension, ischemia/reperfusion damage, cardiac hypertrophy, fibrosis, diastolic dysfunction and heart failure (139). Inflammation and oxidative stress are closely related to each other since inflammatory cells secrete different reactive species resulting in oxidative stress, whereas reactive species can induce intracellular signaling response leading to the expression of pro-inflammatory genes (140). The role of mitochondria in modulating oxidative stress is well-known but recent evidences indicated mitochondria as key players also in the regulation of inflammation (141). During tissue injuries and cell death, damaged mitochondria release biomolecules as endogenous danger-associated molecular patterns (DAMPs), among them reactive oxygen species (ROS) and mitochondrial (mt)DNA fragments (141). DAMPs can trigger an immune response that leads to the activation of neutrophils, T-lymphocytes, macrophages and the imbalance of T helper cytokine profile, thus resulting in a complex inflammation process culminating into fibrosis, arrhythmia, and sudden cardiac death (142, 143), typical features of ACM. Myocardial inflammation and cardiac dysfunction induced by mitochondrial ROS (mtROS) was attenuated by the administration of mtROS specific antioxidant improving mitochondrial function, reducing tissue inflammation and enhancing cardiac function (144).

Recent studies demonstrated a potential link between mtROS and mtDNA with molecular pathways previously associated with ACM pathogenesis. Specifically, mtROS can directly promote differentiation of cardiac fibroblasts into myofibroblasts and extracellular matrix (ECM) deposition by activation of TGF-β1 expression (the most well-known pro-fibrotic cytokine involved in cardiac fibrosis) (145, 146), whereas aberrant mtDNA can stimulate the activation of p38 mitogen-activated protein kinase (MAPK) signaling cascade (147). Interestingly, both pathways are associated with increased expression of both inflammatory and fibrotic genes in ACM *in vitro* model of NRVMs with *Pkp2* knockdown (64), as described above in this review.

Although numerous pieces of evidence concur to a role of oxidative stress in fibrosis, its implication in ACM fibrotic remodeling still has to be investigated. An important independent fibrosis cofactor in ACM hearts is inflammation. A recent study hypothesized a novel mechanistic pathway that links myocyte cell death and myocardial inflammation induced by endurance exercise with mitochondrial dysfunction in *Dsg2*^*mut*/*mut*^ mice (130). Specifically, chronic physical activity in *Dsg2*^*mut*/*mut*^ mice induced the activation of Ca^2+^-dependent cysteine protease calpain-1 (CAPN1) and its association with mitochondria, leading to the cleavage of mitochondrial-bound apoptosis-inducing factor (AIF) (130). Nuclear translocation of truncated AIF in *Dsg2*^*mut*/*mut*^ myocytes triggered large-scale DNA fragmentation and cell death, also enhanced by mitochondrial AIF oxidation due to abundant myocardial ROS and reduced mtROS buffering.

Despite these studies, it is currently unclear whether the activation of these pathways is actually preceded by mitochondrial dysfunction and higher ROS levels in ACM. Up to now, the role of mitochondrial biology in the ACM pathogenesis remains understudied.

Exploring the link between inflammatory pathogenesis of ACM and mitochondrial dysfunction has emerged as a novel interesting topic in order to identify new molecular mechanisms and alternative therapeutic targets as anti-oxidant treatment.

## Molecular Interactions Between Inflammatory Pathways and ACM Pathogenesis

The major molecular mechanism involved in the pathogenesis of ACM is the disruption of mechanical integrity at cell-cell junctions due to defects in the desmosomes. This leads to structural and functional impairment, including abnormal ion channels distribution and gap junction remodeling with consequent electrical abnormalities, impaired protein trafficking, and mitochondrial and calcium dysregulation (8). However, proteins of the desmosome play also a role in transduction pathways, so that desmosomal disarray can result in dysregulation of signaling cascades involved in ACM pathogenesis. The activation of TGF-β pathway in the presence of desmosomal mutations has been proposed as a possible mechanism for the loss of CMs, the accumulation of fibrotic tissue and inflammation observed in ACM (64, 68). Moreover, the suppression of Wnt/β-catenin signaling (148) linked to increased level of GSK3β (69), and activation of Hippo pathway (149) have been associated with the pathological remodeling observed in ACM patients, including activation of pro-apoptotic and inflammatory process, myocyte death and accelerated epithelial to mesenchymal transition that leads to fibro-fatty deposition. Importantly, free plakoglobin can compete with β-catenin in the transcriptional regulation of the Wnt signaling cascade through T-lymphocyte, enhancing binding transcription factors (150). In addition, the activation of NFκB signaling pathway secondary to desmosomal disruption has been identified in different *in vivo* and *in vitro* experimental models, carrying different deleterious desmosomal variants as previously illustrated in details in this review (63). This pathway is a master regulator of cellular inflammatory responses that directly links to the GSK3β pathway based on the fact that activation of GSK3β promotes inflammation through NFκB (151). The evidence of NFκB signaling as inflammatory pathways directly implicated in ACM pathogenesis has been also corroborated by other studies in a mouse model (152) and hiPSCs (153) carrying the deleterious TMEM43-p.S358L variant. The specific activation of NFκB pathway in *Tmem43* knock-in mouse model directly drove the expression of profibrotic TGF-β1 and the subsequent downstream signal cascade, leading to the typical ACM hallmarks as cardiomyocyte death and fibro-fatty substitution (152). Finally, hiPSCs bearing the p.S358L mutation also showed contractile dysfunction that was partially restored after GSK3β inhibition (153), providing solid evidence that targeting NFκB signaling might be beneficial also to restore electrical abnormalities. In addition to NFκB pathway, other inflammatory signaling cascades have been identified by proteomic analysis on human explanted hearts from ACM patients compared to DCM or control hearts (154). Specifically, JAK/STAT3 and ERK pathways were specifically upregulated in the ACM but not in DCM samples, thus suggesting a specific activation of those pathways in cardiac tissue of ACM patients. Also, most of the complement system proteins C3, C4, C6, C7, C8, and C9 were significantly upregulated in ACM samples compared to DCM, suggesting that the complement system activation might be a characteristic molecular phenotype of ACM and not just a common inflammatory heart failure pathway (154). A significant complement activation in the cardiac tissues of patients with ACM was also confirmed by Mavroidis and colleagues (155). Specifically, they also showed a massive complement activation in the myocardium of desmin-null mouse (Des^−/−^) in areas of necrotic cells debris and inflammatory infiltrate and they demonstrated that the inhibition of complement systems activation significantly reduced myocardial remodeling and improved cardiac function and arrhythmias (155).

In conclusion, these findings indicate that specific inflammatory pathways can be considered causative mechanisms related to cardiac degeneration, fibro-fatty substitution and arrhythmia, specifically in ACM pathogenesis. However, molecular mechanisms of inflammatory pathways remain superficial and mainly associated to animal models, thus requiring more in-depth studies for the translation to human disease.

## Discussion

Many questions about ACM pathophysiological mechanisms remain unanswered.

**Figure 2:**
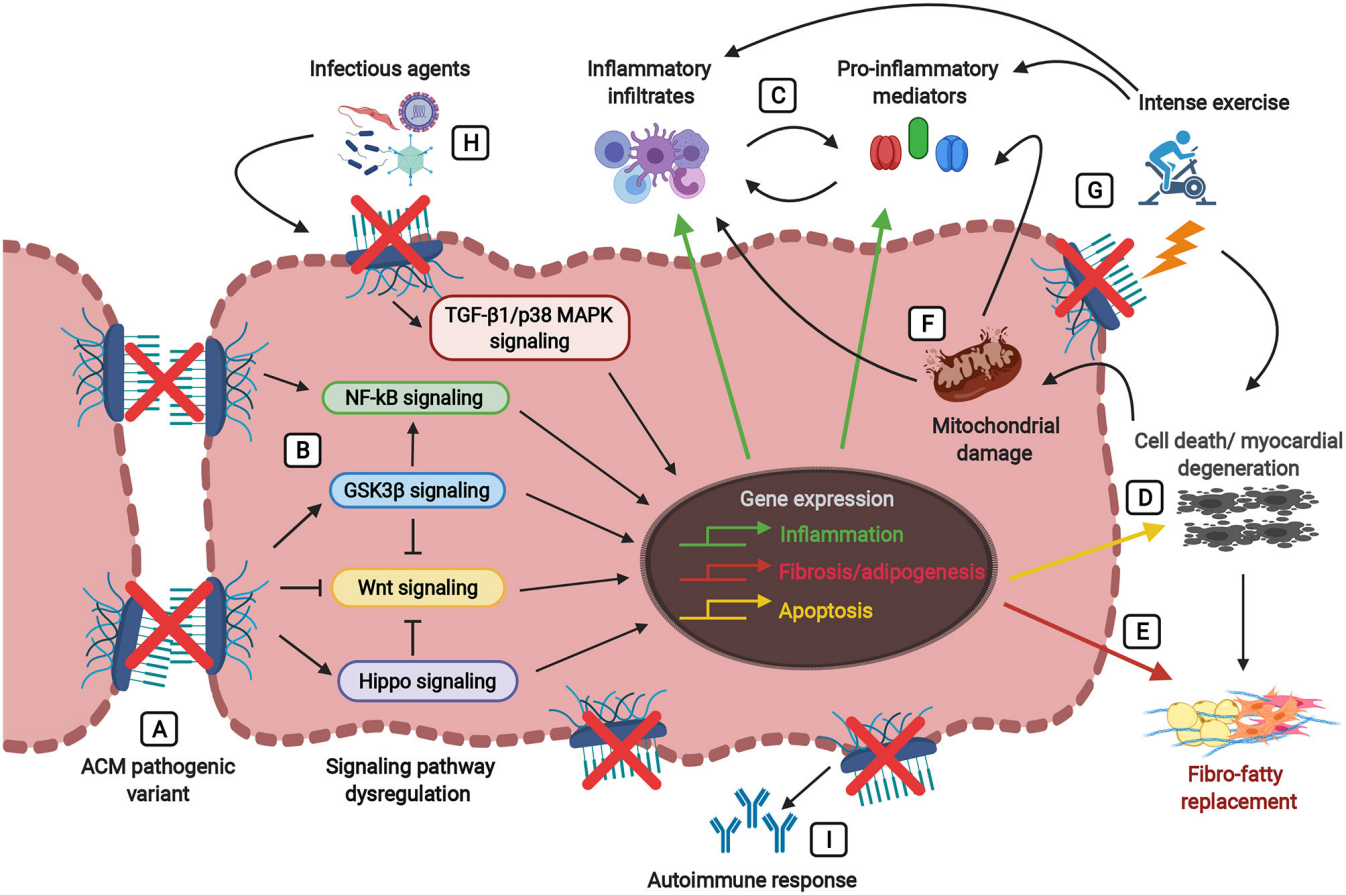
Players involved in the inflammatory theory of ACM pathogenesis, ultimately leading to fibro-fatty replacement. Defects in the desmosomes caused by pathogenic mutations **(A)** trigger signaling cascades leading to the dysregulation of the Wnt pathway (inhibition) and the Hippo, GSK3β, NFκB, and TGF-β1/p38 MAPK pathways (activation) **(B)**. Abnormalities in these signaling pathways lead to the activation of inflammatory, apoptotic, and fibrotic/adipogenic genes, thus resulting in the secretion of inflammatory cytokines (i.e., TNF-α, IL-1, IL-6, IL-8, IL-17) and the infiltration of inflammatory cells (i.e., T-lymphocytes, neutrophils, macrophages, mast cells) **(C)** and cellular damage associated with cardiac cell death **(D)** and fibro-fatty replacement **(E)**. During tissue injury and myocardial degeneration, damaged mitochondria release danger-associated molecular patterns (DAMPs, as reactive oxygen species (ROS) and mitochondrial (mt)DNA fragments), thus further precipitating the immune responses and perpetuating inflammation, apoptosis and fibro-adipogenesis **(F)**. Physical stress due to strenuous exercise not only causes mechanical injury and cell death in the presence of damaged desmosomes, but also stimulates the activation of the immune response and the production of pro-inflammatory mediators **(G)**. The myocardium carrying pathogenic alterations in genes encoding desmosomal proteins is more vulnerable to the presence of infectious agents (i.e., cardiotropic viruses, bacteria, protozoan), leading to the amplification of myocardial inflammation, severe myocyte damage and subsequent precipitation of the disease **(H)**. Desmosomal disruption and myocardial damage might stimulate an immune response by the production of auto-antibodies such as anti-DSG2 (desmoglein 2), AHA (anti-heart), and AIDA (anti-intercalated disk) antibodies **(I)**.

Inflammation in ACM is a multifaceted process involving different players as immune cell infiltrates, infectious agents, inflammatory cytokine secretion, autoimmune antibodies and it can be mediated by mitochondria dysfunction and triggered by physical exercise (Figure 2). The inflammatory process might also modulate the disease severity.

Although growing evidence demonstrated inflammation as an intrinsic feature of ACM, it remains controversial whether inflammation and/or infection are primary events promoting arrhythmia and myocardial damage by immune mechanisms or whether inflammatory process is a secondary reaction in response to progressive myocyte cell death. Studies using transgenic mice carrying deleterious alterations in desmosomal genes demonstrated that the recruitment of inflammatory cells followed the structural damage of myocardium due to progressive myocyte loss initiated by massive cell death (68, 70), thus suggesting myocyte necrosis as crucial initiator of myocardial injury triggering inflammatory process as secondary response.

On the other hand, exposure to pro-inflammatory cytokines was sufficient to induce abnormal internalization of plakoglobin, mimicking the junctional instability and desmosomal disruption observed in ACM patients (40). Myocardial inflammation in cardiac tissues (40) and hiPSC-derived CMs (63) from ACM patients carrying desmosomal deleterious alterations has been observed even in absence of inflammatory cell infiltration. Moreover, a recent work suggested a relation between abundance of *Pkp2* transcripts and the activation of inflammatory and immune pathways endogenous to adult *Pkp2*-knock out cardiomyocytes, even without pathogens as external triggers (75). These findings demonstrate that cardiomyocytes themselves can activate an immune response and produce inflammatory mediators, thus indicating a direct contribution of myocytes to inflammatory process independently of cardiac tissue degeneration and recruitment of specialized inflammatory cells as main triggers. In this view, inflammation would act as a driver instead of as simple modulator. It is worth noting that ACM is now starting to be rewarded as a multicellular or even multi-organ disease (156), which would support an active role for the (mutated) innate immune cells.

A comprehensive analysis of the ACM inflammasome is still lacking in both animal models and patients and there is a urgent need to systematically investigate ACM inflammation process in particular dissecting the different—concealed, overt, and end—phases of the disease.

A complete characterization of the cross-talk among different inflammatory mediators might distinguish an ACM-specific inflammatory cascade (157), improving the understanding of the immune response in ACM and identifying potential new therapeutic targets to limit inflammatory process in ACM patients. For example, the identification of autoantibodies in ACM opens novel promising therapeutic opportunities, including immune therapies, similar to those proposed for autoimmune *pemphigus vulgaris* (158) and virus-negative myocarditis/inflammatory dilated cardiomyopathy (159).

As additional note, expanding the knowledge of inflammation-related pathogenic mechanisms in ACM might provide new insights to improve diagnosis and risk stratification, as well as to develop novel therapeutic opportunities by targeting immune signaling. As promising new clinical perspective, anti-oxidant (144), anti-inflammatory treatments (160), and immunosuppressive therapy (161) might be beneficial to reduce myocardial damage and arrhythmia in ACM patients, as recently observed for other cardiac diseases.

Altogether, the studies we have reviewed here collectively support a significant contribution of inflammation to ACM pathogenesis, either as active driver or as modulator of the disease phenotype.

It is tempting to speculate that inflammation in ACM acts with both a primary and secondary role, depending on the underlying genotype (gene mutation or even mutation-specific), or on the different clinic-pathological phases (pre-histological, preclinical, pre-symptomatic, and symptomatic), or even on the interaction between genetic background and environment (e.g., gene modifiers, but also physical activity, psychological stress). Further mechanistic studies are still required to dissect when inflammation acts as active driver and when as secondary event in the pathogenesis of ACM, so that mechanistic-targeted therapies can be developed.

## Author Contributions

VM, MA, OC, and MB outlined, drafted, and contributed to the writing of the manuscript. All authors approved the final version of the manuscript.

## Funding

This work was supported by the European Research Area Network on Cardiovascular Diseases (ERA-CVD), Transnational Research Project on Cardiovascular Diseases (JTC2016_FP-40-021 ACM-HF), the Netherlands Organisation for Health Research and Development ZonMW (MKMD project no. 114022504), the European Research Council (ERC-CoG Mini-HEART no. 101001746) and by Sociedad Española Cardiología, Proyecto Investigación Básica Cardiología 2020 (SEC/FEC-INV-BAS 20/003).

## Conflict of Interest

The authors declare that the research was conducted in the absence of any commercial or financial relationships that could be construed as a potential conflict of interest.

## Publisher's Note

All claims expressed in this article are solely those of the authors and do not necessarily represent those of their affiliated organizations, or those of the publisher, the editors and the reviewers. Any product that may be evaluated in this article, or claim that may be made by its manufacturer, is not guaranteed or endorsed by the publisher.
